# Decrease of Functional Activated T and B Cells and Treatment of Glomerulonephitis in Lupus-Prone Mice Using a Natural Flavonoid Astilbin

**DOI:** 10.1371/journal.pone.0124002

**Published:** 2015-04-13

**Authors:** Lele Guo, Wen Liu, Tingting Lu, Wenjie Guo, Jian Gao, Qiong Luo, Xuefeng Wu, Yang Sun, Xudong Wu, Yan Shen, Qiang Xu

**Affiliations:** State Key Laboratory of Pharmaceutical Biotechnology, School of Life Sciences, Nanjing University, 22 Hankou Road, Nanjing 210093, China; Radboud university medical center, NETHERLANDS

## Abstract

Treatment of systemic lupus erythematosus (SLE), a chronic inflammatory disease, involves the long-term use of immunosuppressive agents with significant side effects. New therapeutic approaches are being explored to find better treatment possibilities. In this study, age-matched female MRL/lpr mice were treated orally with a natural flavonoid astilbin. Astilbin administration started either at week 8 or week 12 of age though week 20. In the early treatment regimen, the treatment with astilbin reduced splenomegaly / lymphomegaly, autoantibody production and ameliorated lupus nephitis. Several serum cytokines were significantly decreased upon treatment including IFN-g, IL-17A, IL-1b, TNF-a and IL-6. Both spleen CD44^hi^CD62L^lo^ activated T cells and CD138^+^B220^-^ plasma cells greatly declined. Furthermore, astilbin treatment resulted in decreased mitochondrial membrane potential in activated T cells and downregulated expression of the co-stimulatory molecules CD80 and CD86 on LPS stimulated B cells. Similar but less profound effectiveness was observed in the mice with established disease in the late treatment regimen. These results indicate that the natural product astilbin can mitigate disease development in lupus-prone mice by decreasing functional activated T and B cells.

## Introduction

Systemic lupus erythematosus (SLE) is a systemic autoimmune disease with high titer production of autoantibodies, several cytokine aberrations and clinical involvement in multiple organ systems [1]. SLE is best characterized by the presence of activated T and B cells. T cells from SLE patients have an altered pattern of gene expression that modifies their behavior and grants them increased inflammatory capacity [2]. They also provide help to autoreactive B cells. On the other hand, SLE B cells are not merely the passive producers of autoantibodies, but play a central role in autoimmunity via nonconventional mechanisms, including autoantigen presentation to T cells [3].

Current therapies for SLE are not ideal in terms of efficacy and toxicity even though intensive research has increased our understanding of the pathogenesis of SLE [4–6]. Indiscriminate immunosuppressive drugs such as cyclophosphamide (CTX), azathioprine and mycophenolate mofetil are very toxic, and only 50% of treated patients enter complete remission, with relapse rates up to 30% over a 2-year period [7]. Specific targeted therapies including T or B cell-targeted therapy have been developed, but the clinical benefit is modest, even controversial. B-cell depleting therapies in three separate, placebo-controlled, phase III trials using anti-human CD20 mAbs, rituximab and ocrelizumab, failed to show benefit, and safety was compromised as opportunistic infections increased with treatment over time [8]. Inhibition of T cell activation using anti-CD40L antibodies or CTLA4Ig (abatacept) was not safe or effective in lupus patients [9, 10]. Thus, SLE is a clinically and immunologically heterogeneous disease and novel therapies aiming at higher treatment efficacy and fewer adverse effects are being explored.

Astilbin, a natural flavonoid, which can be extracted from the medicinal herbs Smilacx glabra, is able to interfere with different biological processes [11–13]. Our previous work demonstrates that astilbin can selectively induce apoptosis in activated but not nonactivated T cells, stimulate negative regulatory cytokine interleukin-10 and suppress activated T-cell adhesion and migration [14–18]. Other colleagues have reported that astilbin inhibits maturation and function of dendritic cells, and induces TGF-β and IL-10 production by these cells [19, 20]. Such a natural product with immunomodulating activity shows significantly effective in many animal models for immune diseases including rheumatoid arthitis, contact dermatitis and immunologic liver injury, but without obvious adverse effects even after long-term oral administration [15, 21, 22].

In this study, astilbin was found to delay the disease development in lupus-prone MRL/lpr mice when preventive oral administration was started before the onset of disease and also when disease onset preceded the initiation of treatment. Astilbin treatment reduced circulating anti-nuclear antibodies, several serum cytokines and resulted in a dramatic decrease in functional activated T and B cells. These results suggest the potential therapeutic utility of the natural flavonoid astilbin in the management of early-stage and progressive manifestations of SLE.

## Materials and Methods

### Agents

Astilbin, 3, 3, 4′, 5, 7-pentahydroxyflavanone 3–6[-deoxy-([alpha]-L-mannopyranoside)], was isolated from the rhizome of *Smilax glabra* and purified in our laboratory as previously described [11, 23]. The purity was determined by HPLC to be above 99% (S1 Fig). CTX was purchased from Jiangsu Hengrui Medicine Co., Ltd. LPS and resveratrol were purchased from Sigma (St Louis, MO).

### Mice and treatment

Female MRL/MpJ-Tnfrsf6^lpr^ (MRL/lpr) mice were purchased from The Experimental Animal Center of the Academy of Military Medical Science (Beijing, China). They were maintained with free access to pellet food and water in plastic cages at 21 ± 2°C and kept on a 12 h light/dark cycle. Genotyping and sequence confirmation were performed by PCR analyses of tail genomic DNA. About 65 MRL/lpr mice were randomized into the following treatment regimens by using computer-generated random numbers. In the early treatment regimen, 8-week-old MRL/lpr mice were orally administered with astilbin in 0.5% methylcellulose at 10, 20 or 40 mg/kg every other day (n = 8, 14, 8 mice per group, respectively). Vehicle was provided orally as 0.5% methylcellulose only (n = 10 mice). CTX was provided intraperitoneally as standard of care at 20 mg/kg every 6 days (n = 10 mice). In the late treatment regimen, the efficacy of astilbin in the established disease was evaluated. Diseased MRL/lpr mice were screened by monitoring the presence of serum anti-nuclear antibodies (ANA) and degree of proteinuria every week. Vehicle and astilbin (20 mg/kg) administration was initiated from week 12 of age (n = 6, 8 mice per group, respectively), at which time proteinuria was detected positive (30–300mg/dL) by a urine stick assay (Albustix, Siemens). Mice were sacrificed under sodium pentobarbital anesthesia at 20 wk in both treatment regimens except that some of mice were maintained until 30 wk for determination of long-term effects. Animal welfare and experimental procedures were carried out in accordance with the Guide for the Care and Use of Laboratory Animals (Ministry of Science and Technology of China, 2006) and the related ethical regulations of our university. All the animal experiments were approved by Nanjing University Animal Care and Use Committee (NJU-ACUC) and made to minimize suffering and to reduce the number of animals used.

### Histology

The left kidney from each animal was removed and fixed in 10% buffered formalin for 30 h. Specimens were then gradually dehydrated in alcohol and embedded in paraffin. Kidney stains used for histological analyses included H&E, periodic acid–Schiff (PAS) and trichome stains. Only H&E-stained sections are shown in this report; however, all stained sections were evaluated in a blinded fashion by a pathologist for scoring purposes. Multiple stains were used to determine score values. The method used for renal histopathological assessment was as follows [24]: glomerular cellularity, glomerular necrosis, glomerulosclerosis, tubular epithelial degeneration, tubular atrophy, interstitial infiltration and vasculitis were all given a score of 1–5 for null, low, moderate, high, or severe disease, respectively.

### Assessment of anti-nuclear antibodies

Serum ANA titers were determined by monitoring binding of serially diluted samples to HEp-2 slides (EUROIMMUN, Lübeck, Germany). Sera were added for 30 min at 37°C in a moist chamber. The slides were then washed extensively in phosphate buffered saline (PBS), incubated for 30 min at 37°C with a fluorescein isothiocyanate conjugated anti-mouse immunoglobulin (DAKO A/S, Glostrup, Denmark), and examined by fluorescence microscopy (Olympus, Japan). Specific fluorescence at a serum dilution of ≥ 1:40 was considered positive [24]. Serum levels of anti-dsDNA autoantibodies were measured with specific ELISA kit following the manufacturer's instruction (Alpha Diagnostic, San Antonio, Texas).

### Immunofluorescence evaluation of C3 deposits in kidney

Paraffin-embedded kidney sections were stained with a fluorescein isothiocyanate conjugated rabbit polyclonal antibody against complement C3c (Abcam, Cambridge, MA), and examined by fluorescence microscopy. DAPI was used to stain the cell nuclei.

### Cytokine analysis by CBA assay

Serum levels of IFN-γ, IL17A, IL-1β, TNF-′ and IL-6 were determined using Cytometric Bead Array (CBA) cytokine assay kit according to the manufacture as recommended by BD pharmingen (Franklin Lakes, NJ).

### Flow cytometry

Single-cell suspensions of splenocytes and lymph node cells were treated with RBC lysis buffer (Sigma), and washed with FACS buffer (PBS plus 1% FBS). These cells were incubated with specific surface-binding antibodies for 30 min at 4°C. Antibodies included anti-mouse CD3e-percp-cy5.5, anti-mouse CD19-FITC, anti-mouse CD44-FITC, anti-mouse CD62L-PE, anti-mouse B220-APC, anti-mouse CD80-APC, anti-mouse CD86-APC and rat IgG_1_-APC isotrol control and rat IgG_2a_-PE isotrol control (all from eBioscience, San Diego, CA), anti-mouse CD138-PE (Miltenyi Biotech, Bergisch Gladbach, Germany), anti-mouse CD4-APC, anti-mouse CD8-APC (BD pharmingen). In some experiments, splenocytes were stimulated with LPS (1 μg/ml) or PBS for 24 h before stained with fluorochome-conjugated CD80, CD86 and CD19 antibodies. For JC-1 staining, splenocytes were stimulated with anti-CD3 and anti-CD28 antibodies (1 μg/ml for each, BD pharmingen) for 24 h and then incubated with 5 μg/ml JC-1 (Biotium, Hayward, CA) for 30 min at 37°C. Samples were analyzed by flow cytometry on a FACScan flow cytometer (Becton Dickinson).

### Quantitative PCR

RNA samples isolated from spleens were reverse transcribed to cDNA and subjected to quantitative PCR, which was performed with the BioRad CFX96 Touch^TM^ Real-Time PCR Detection System (BioRad, Hercules, CA) using iQ^TM^ SYBR Green Supermix (BioRad), and theshold cycle numbers were obtained using BioRad CFX Manager software. The program for amplification was 1 cycle of 95°C for 2 min followed by 40 cycles of 95°C for 10 s, 60°C for 30 s. The primer sequences used in this study were as follows: IFN-γ, 5′-GCAGCCAACCTAAGCAAGAT-3′ (forward), and 5′-GGGTCACCTGACACATTCAA-3' (reverse); TNF-′, 5'-CGAGTGACAAGCCTGTAGCCC-3′ (forward) and 5′-GTCTTTGAGATCCATGCCGTTG-3′ (reverse); IL-17A, 5′-TCGAGAAGATGCTGGTGGGT-3′ (forward) and 5'-CTCTGTTTAGGCTGCCTGGC-3′ (reverse); BAFF, 5′-TGTTGTCCAGCAGTTTCAC-3′ (forward), and 5′-CTGCAGACAGTCTTGAATGA-3′ (reverse); APRIL, 5'-ACTCTCAGTTGCCCTCTGGTTG-3′ (forward), and 5'-GGAACTCTGCTCCGGGAGACTC-3′ (reverse); β-actin, 5'-GTATGCCTCGGTCGTACCA-3′ (forward), and 5'-CTTCTGCATCCTG-TCAGCAA-3′ (reverse).

### Statistical analysis

Data are expressed as means ± SEM from at least three independent experiments. Statistical significance between groups was measured using Mann-Whitney’s U test for assessment of ANA titers and two-tailed Mann-Whitney’s t test for renal histopathological assessment, respectively. In other experiments, one-way analysis of variance (ANOVA), followed by Student two-tailed t test, was applied to evaluate the significance of differences between groups. *p* < 0.05 was considered to be significant.

## Results

### Prevention of disease development by oral administration of astilbin to MRL/lpr mice

**Fig 1:**
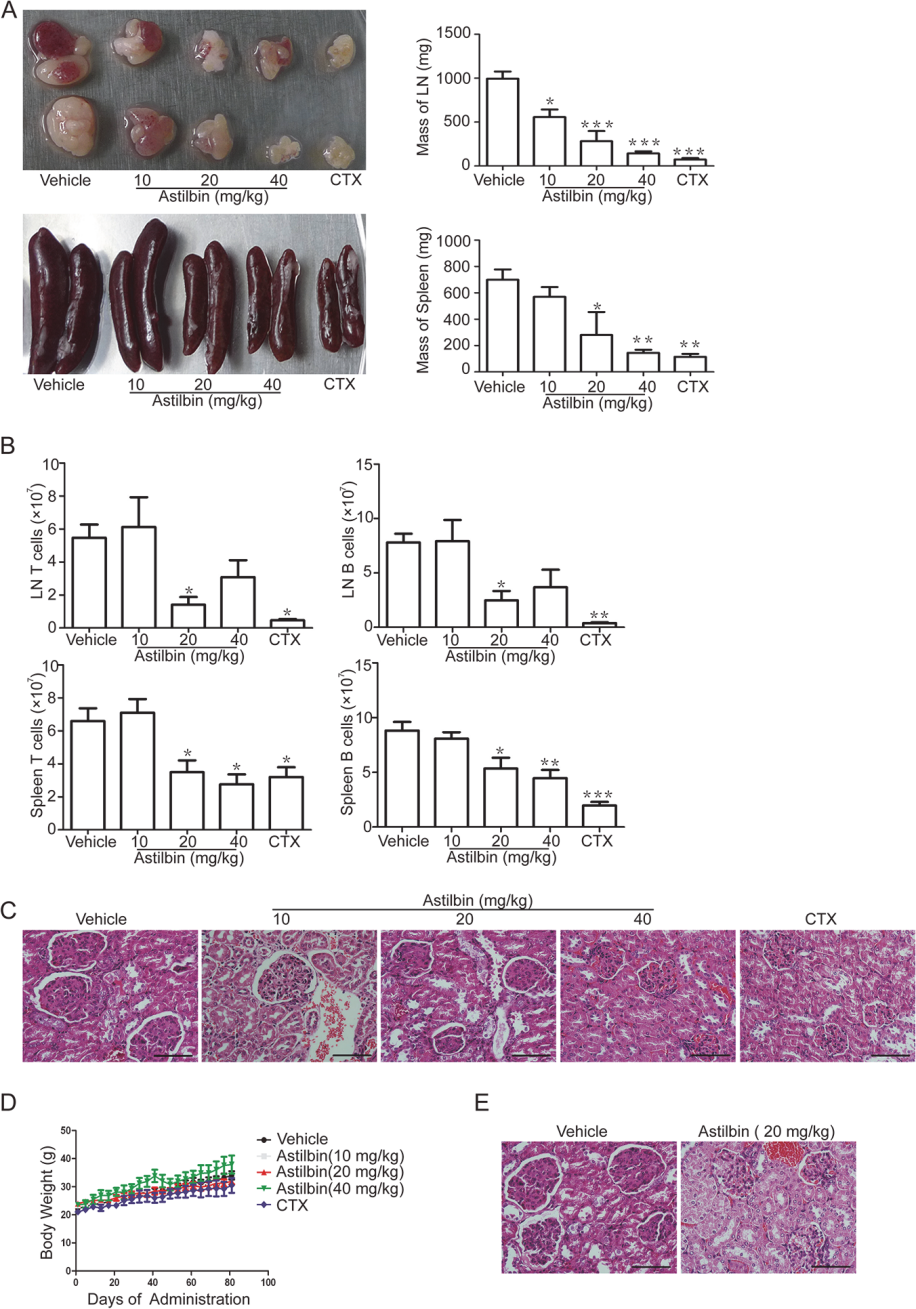
Treatment of MRL/lpr mice with astilbin ameliorates disease development associated with lupus. **A.** Representative lymph nodes and spleen from MRL/lpr mice treated with astilbin or CTX starting at 8 weeks of age (left panel). The lymph nodes and spleen from treated mice were weighed and graphed as mean ± SEM (right panel). **B.** Total number of CD3^+^ T and CD19^+^ B cells in the lymph nodes (LN) and spleen. The absolute cell number was obtained by multiplying the total splenocyte or lymph node cell number by the percentage of CD3^+^ T and CD19^+^ B cells. **C.** H&E-stained paraffin wax-embedded kidney sections from treated MRL/lpr mice at 20 wk. Bar equals to 50 μm. Representative images shown from n = 5–10 samples were later scored, with results displayed in Table 1. **D.** Body mass for each individual mouse measured over time graphed as mean ± SEM from 8 to 20 weeks of age. **E.** H&E-stained kidney sections at 20 wk from MRL/lpr mice treated with astilbin starting from 12 weeks of age. Bar equals to 50 μm. The data show mean ± SEM, n ≥ 6 mice per group as described in Materials and methods. * *p* < 0.05, ** *p*< 0.01, *** *p* < 0.001 versus vehicle controls.

Astilbin was orally administered at 10, 20 and 40 mg/kg to MRL/lpr mice starting at 8 wk of age. As a control, CTX was administered by the intraperitoneal injections at a dose of 20 mg/kg. By 20 wk of age, astilbin-treated mice exhibited significantly reduced lymphomegaly and splenomegaly at both 20 and 40 mg/kg but not at 10 mg/kg (Fig 1A). Reduced CD3^+^ T and CD19^+^ B cell numbers in both lymph nodes and spleens were also observed at 20 mg/kg dose compared with vehicle (Fig 1B). End-stage SLE nephitis was evaluated by histopathology and scored by a board-certified pathologist for the assessment of total renal damage in diseased animals. A decrease in glomerular size was noted in H&E-stained renal sections from two astilbin treatment groups (20 and 40 mg/kg) compared with vehicle treatment group (Fig 1C). At the highest dose of astilbin, significant reductions in histopathology scores relative to vehicle controls were reported for glomerular cellularity, glomeruloscleosis and vasculitis (Table 1). The rate of body weight gain did not change significantly for any group tested (Fig 1D). But severe alopecia was observed in CTX treatment group (data not shown). To investigate whether astilbin administration could prevent the ongoing development of the lupus-like disease in MRL/lpr mice, the start of treatment was delayed until 12 wk of age (after the onset of disease defined by both ANA and proteinuria positive). At 20 wk, histological analysis displayed the mildly decreased degree in glomerular size upon treatment with 20 mg/kg of astilbin (Fig 1E).

**Table 1:** Effects of astilbin treatment on renal histological scores.

Independent Histopathologist Scores (Scores Grade: 0–5)							
MRL/lpr SLE Model Group	Glomerular Cellularity	GlomerularNecrosis	Glomerulosclerosis	Tubular Epithelial Degeneration	Tubular Atrophy	Interstitial Infiltration	Vasculitis
Vehicle (n = 8)	3.75 ± 0.50	1.75 ± 0.96	3.50 ± 0.58	3.50 ± 0.58	1.75 ± 0.68	2.00 ± 0.82	2.67 ± 0.58
Astilbin 10 mg/kg (n = 5)	3.00 ± 0.00	1.67 ± 0.52	3.00 ± 0.00	4.33 ± 0.57	1.00 ± 0.00	1.66 ± 0.52	1.33 ± 0.58 *
Astilbin 20 mg/kg (n = 10)	2.75 ± 0.50	1.50 ± 0.45	2.75 ± 0.57	2.25 ± 0.68	1.00 ± 0.00	1.25 ± 0.50	1.25 ± 0.50 *
Astilbin 40 mg/kg (n = 6)	2.00 ± 0.45 *	1.00 ± 0.00	1.33 ± 0.58 *	3.00 ± 0.78	1.25 ± 0.52	1.00 ± 0.00	1.00 ± 0.00 *
CTX 20 mg/kg (n = 9)	1.66 ± 0.57 *	1.67 ± 0.57	1.66 ± 0.52 *	3.66 ± 0.58	1.00 ± 0.00	1.00 ± 0.00	1.33 ± 0.58 *

Pathologist scored H&E-, PAS-, and trichrome-stained paraffin wax-embedded kidney sections displayed as mean ± SEM. Pathologist scoring method and definition can be found in Materials and Methods. Statistics were performed using a two-tailed Mann–Whitney t test.

*p ≤ 0.05, (compared with vehicle).

**Fig 2:**
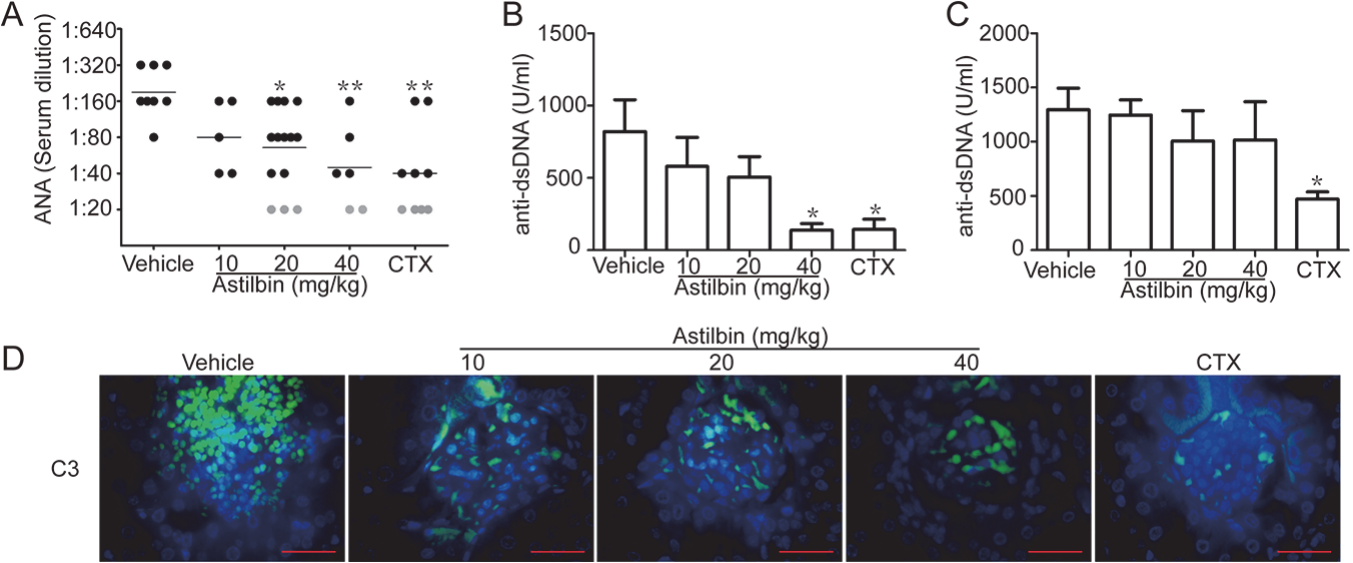
Astilbin treatment reduces production of autoantibodies and complement C3 deposits. MRL/lpr mice were treated with astilbin or CTX starting at 8 weeks of age. Sera were collected from the mice at 20 or 30 wk. **A.** ANA titers at 20 wk. ANA titers are evaluated by scoring serum dilutions (from 1:20 to 1:320) and a higher titer is defined as detectable in more dilution. Each circle indicates the ANA titer of an individual mouse and black circles mean positive response. **B.** Anti-dsDNA Ab levels in sera at 20 wk. n ≥ 6 per group as described in Materials and methods. **C.** Anti-dsDNA Ab levels in sera at 30 wk. n = 3 mice per group except astilbin treatment group (20 mg/kg, n = 4). The serum levels of anti-dsDNA antibodies were determined by ELISA. The data show mean ± SEM. * *p* < 0.05, ** *p*< 0.01 versus vehicle controls. **D.** C3 deposits in the kidney sections from treated MRL/lpr mice at 20 wk. C3 deposits were detected by immunefluorescence microscopy. Bar equals to 1 μm.

The progressive development of antoantibodies is another of the disease manifestations associated with SLE. In the early treatment regimen, serum antibodies against topoisomerase I, dsDNA and histone were evaluated by indirect immunofluorescent staining of Hep-2 cells. By 20 wk of age, all of the MRL/lpr mice in vehicle treatment group were ANA-positive, whereas the ANA-positive frequency of astilbin-treated mice was 100% (5/5, 10 mg/kg), 78.6% (11/14, 20 mg/kg) and 66.7% (4/6, 40 mg/kg), respectively (Fig 2A). Moreover, the average ANA titer of two astilbin treatment groups (20 and 40 mg/kg) was significantly lower than that of vehicle controls. Similarly, the serum levels of anti-dsDNA Abs was significantly reduced by the treatment of astilbin at 40 mg/kg dose (Fig 2B). By 30 wk of age, no significant decrease in the serum anti-dsDNA Ab level was observed upon astilbin treatment (Fig 2C). These results indicate that early treatment with astilbin slowed and reduced, but did not entirely inhibit, autoantibody production. When Renal sections were stained for detecting complement C3 deposition, immunofluorescence for C3 in the kidney from the mice treated with astilbin showed remarkable reduction compared with that from vehicle controls at 20 wk (Fig 2D).

### Effects of astilbin treatment on proinflammatory cytokine production

**Fig 3:**
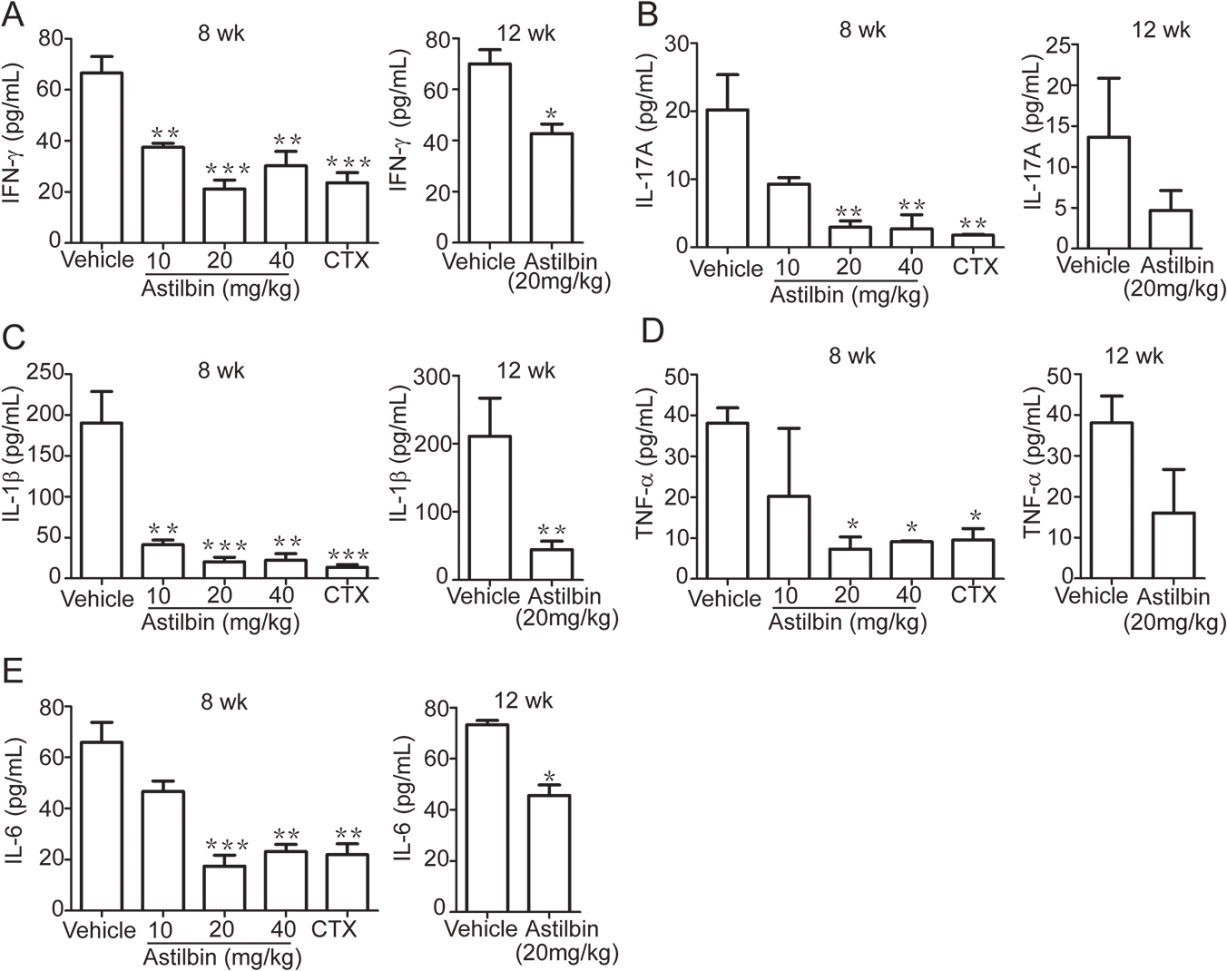
Astilbin treatment reduces proinflammatory cytokine production. MRL/lpr mice were treated with astilbin (10, 20, 40 mg/kg) starting at 8 weeks or astilbin (20 mg/kg) at 12 weeks of age. Sera were collected at 20 wk and serum cytokine levels were determined by CBA assay. **A.** IFN-γ. **B.** IL17A. **C.** IL-1β. **D.** TNF-′. **E.** IL-6. The data show mean ± SEM, n ≥ 6 mice per group as described in Materials and methods. * *p* < 0.05, ** *p*< 0.01, *** *p* < 0.001 versus vehicle controls.

To evaluate whether astilbin treatment causes reduction or modulation in proinflammatory cytokine profiles, serum cytokine levels were detected at 20 wk using CBA assay. In the early treatment regimen starting at 8 wk, both doses (20 and 40 mg/kg) of astilbin significantly reduced IFN-, IL-17A, IL-1β TNF-′and IL-6 compared with vehicle (Fig 3). Of note, these cytokines decreased to the levels of those in CTX treatment group. In the late treatment regimen starting at 12 wk, astilbin administration also caused a significant reduction in the serum levels of IFN-γ, IL-1β and IL-6 (Fig 3A, 3C and 3E). In addition, the delayed astibin administration resulted in a decreasing tendency of the other two cytokine levels (Fig 3B and 3D).

### Decreases in the number and function of activated T cells by astilbin treatment

**Fig 4:**
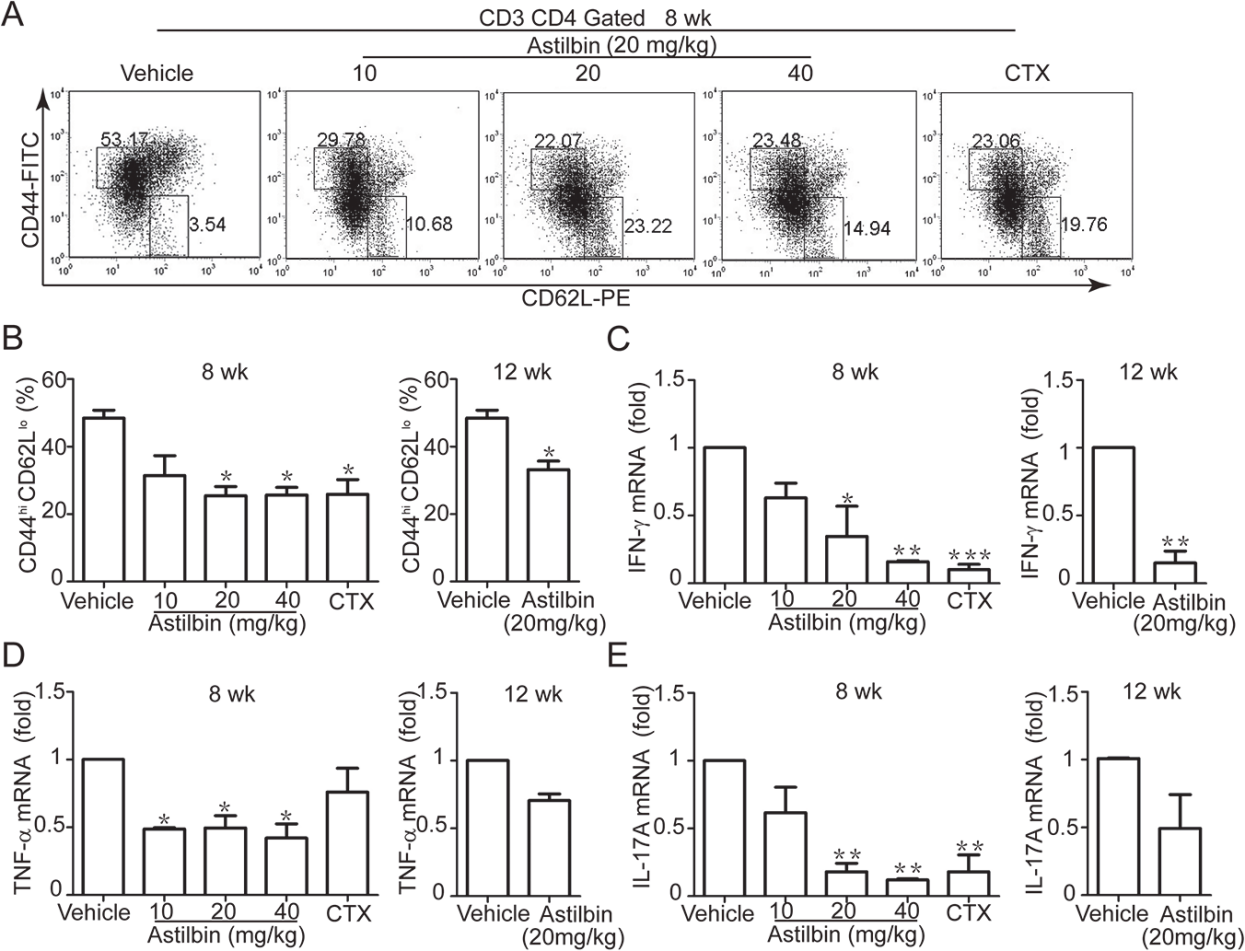
Astilbin treatment decreases the percentage of CD44^hi^CD62L^lo^ activated CD4^+^ T cells and several cytokine mRNA expression. MRL/lpr mice were treated with astilbin (10, 20, 40 mg/kg) starting at 8 weeks or astilbin (20 mg/kg) at 12 weeks of age. Flow cytometry analysis of whole splenocytes stained with CD3e-percp-cy5.5, CD44-FITC, CD62L-PE, CD4–APC was performed at 20 wk. **A.** Representative flow cytometry dot plots of live cell events gated on CD3^+^ CD4^+^ cells. **B.** Graphs show the percentage of CD44^hi^CD62L^lo^ CD3^+^ CD4^+^ T cells in the spleens from the mice treated as indicated. The mRNA expression of IFN- (**C**), TNF-′ (**D**) and IL17A (**E**) in the spleens from the treated mice was examined by real-time PCR. β-actin was used as loading control. The data show mean ± SEM of six mice. * *p* < 0.05,** *p*< 0.01, *** *p* < 0.001 versus vehicle controls.

To determine the mechanism though which astilbin prevents disease development in MRL/lpr mice, we tested the effects of astilbin treatment on the number and function of activated T cells. Activated CD4^+^ T cells accumulate in lupus-prone mice and promote disease development, in part, by providing cognate help for autoreactive B cells [10, 25]. In vehicle-treated MRL/lpr mice, ~50% of CD4^+^ T cells from splenocytes bore an activated phenotype characterized by CD44^hi^ CD62L^lo^ (Fig 4A). A significant decrease in the percentage of activated CD4^+^ T cells was observed for the 20 and 40 mg/kg dose compared with vehicle in the early treatment regimen (Fig 4B).Their frequency was considerable with that of CTX. The delayed treatment of astilbin also caused a significant reduction in this population (Fig 4B). In addition, similar results were obtained for the frequency of activated CD8^+^ T cells (S2 Fig). Given the dramatic reduction in splentic T cells, similar values were recorded for the absolute numbers of activated CD4^+^ and CD8^+^ T cells (data not shown). Furthermore, the early astilbin treatment of 20 and 40 mg/kg doses reduced the mRNA expression of IFN-γ, TNF′ and IL-17A in the spleen to the level of that of the CTX treatment, and only reduction in the IFN-γ mRNA level was detected for the delayed astilbin administration (Fig 4C–4E).

### In vitro effect of astilbin on the mitochondrial membrane potential in activated T cells

**Fig 5:**
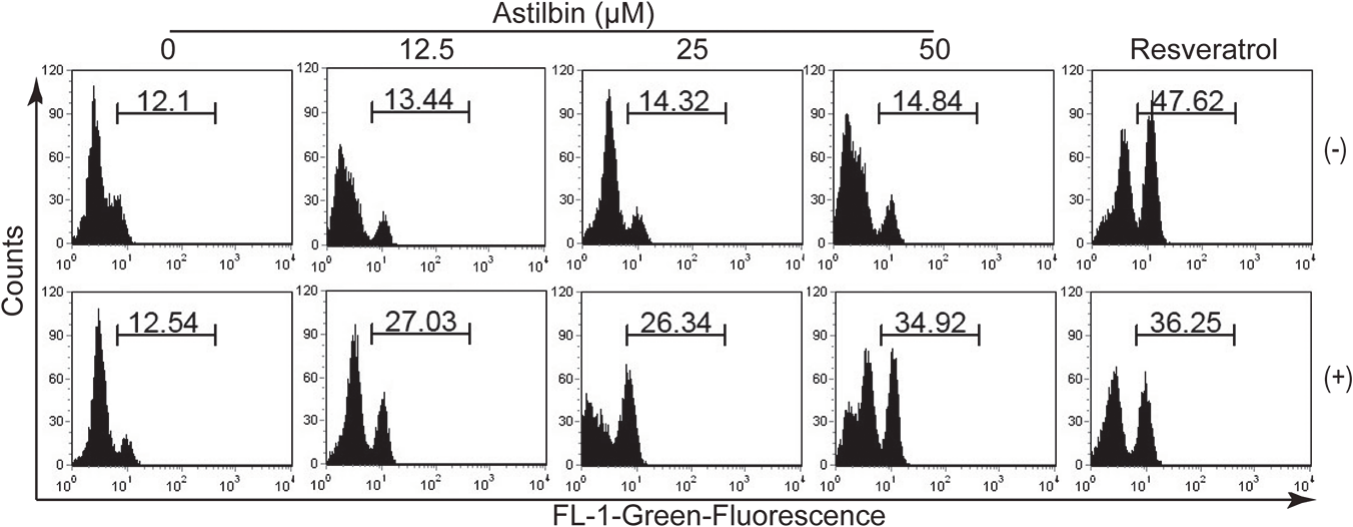
Astilbin decreases the mitochondrial membrane potential in activated T cells. Splenocytes were isolated from 12-week-old naive MRL/lpr mice and stimulated without (-) or with (+) anti-CD3 and anti-CD28 antibodies (1 μg/ml for each) for 24 h. These cells were incubated with various concentrations of astilbin or resveratrol (50 M) for 24h and then stained with JC-1. Flow cytometry analysis was performed. Data were representative of thee independent experiments (n ≥ 3 mice).

Because our previous work demonstrates that astilbin induces apoptosis in activated T cells possibly in a mitochondria-dependent pathway, we measured the change in mitochondrial membrane potential using JC-1, a potential-sensitive dye. The splenocytes from 12-week-old untreated MRL/lpr mice were activated with or without anti-CD3 and anti-CD28 antibodies for 24 h and subsequently incubated with various concentrations of astilbin. As shown in Fig 5, astilbin caused an increase in the green fluorescence of JC-1 in activated T cells, indicating the depolarization of mitochondrial membrane potential. In contrast, no significant increase was observed in astilbin-treated nonactivated T cells. As a positive control, 50 μM of resveratrol resulted in mitochondrial depolarization in both nonactivated and activated T cells.

### Decrease in the number and function of activated B cells by astilbin treatment

**Fig 6:**
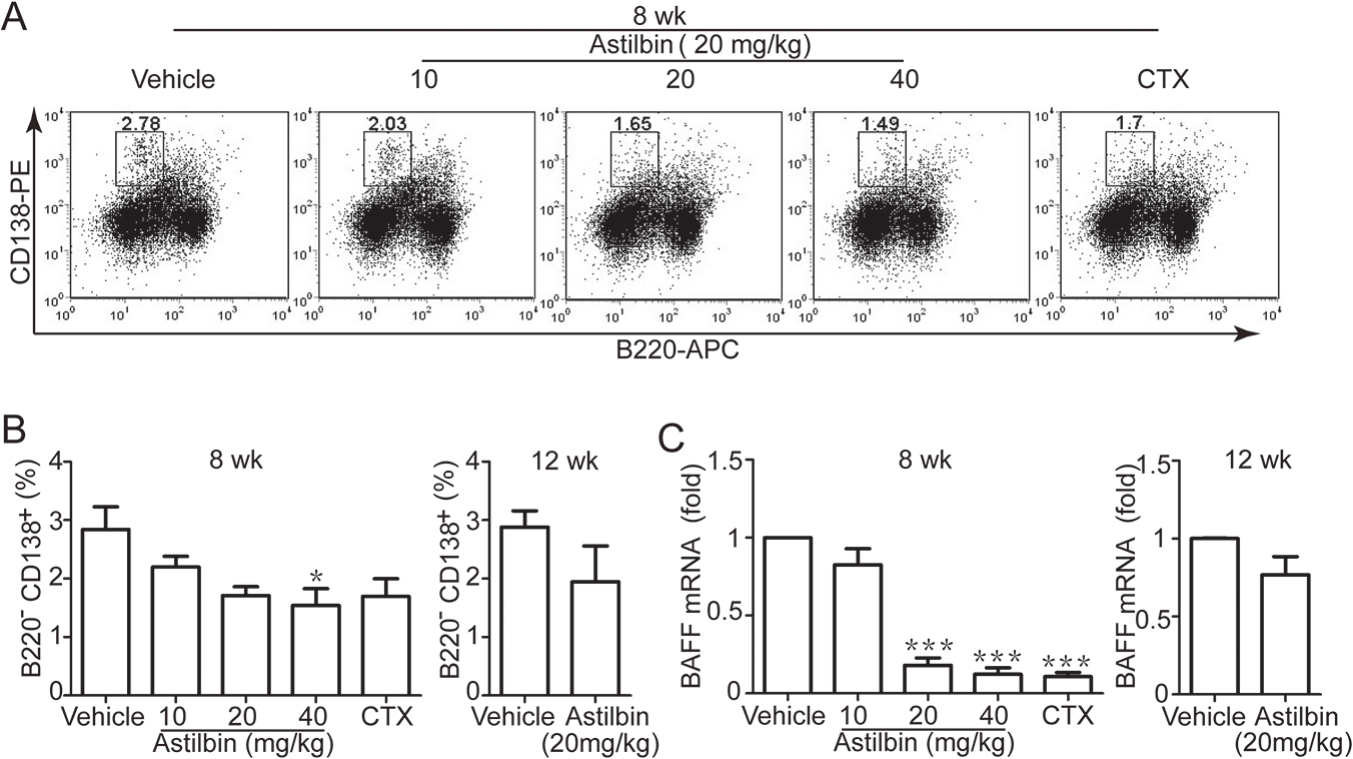
Astilbin treatment decreases the percentage of CD19^-^B220^-^CD138^+^ plasma cells and BAFF mRNA expression. MRL/lpr mice were treated with astilbin (10, 20, 40 mg/kg) starting at 8 weeks or astilbin (20 mg/kg) at 12 weeks of age. Flow cytometry analysis of whole splenocytes stained with CD19-FITC, CD138-PE, B220–APC was performed at 20 wk. **A.** Representative flow cytometry dot plots of live cell events gated on CD19^-^ cells. **B.** Graphs show the percentage of CD19^-^B220^-^CD138^+^ plasma cells in the spleens from the mice treated as indicated. **C.** The mRNA expression of BAFF in the spleens from the treated mice was examined by real-time PCR. β-actin was used as loading control. The data show mean ± SEM of six mice. *p < 0.05, *** *p* < 0.001 versus vehicle controls.

To test the effects of astilbin treatment on the number and function of activated B cells, the percentage of CD19^-^ B220^-^CD138^+^ plasma cells (autoantibody-producing cell types) in the spleens was evaluated. This population in astilbin-treated mice was reduced significantly in the early, but slightly in the late treatment regimen (Fig 6A and 6B). Similar values were recorded for their absolute numbers (data not shown). Furthermore, the early astilbin administration resulted in a dramatic decrease in the mRNA level of B cell activating factor (BAFF) in the spleens at 20 and 40 mg/kg compared with vehicle (Fig 6C). The inhibitory effect of two doses of astilbin was considerable with that of CTX. There was a slight but less profound decrease in BAFF mRNA expression when astilbin administration was initiated at 12 wk.

**Fig 7:**
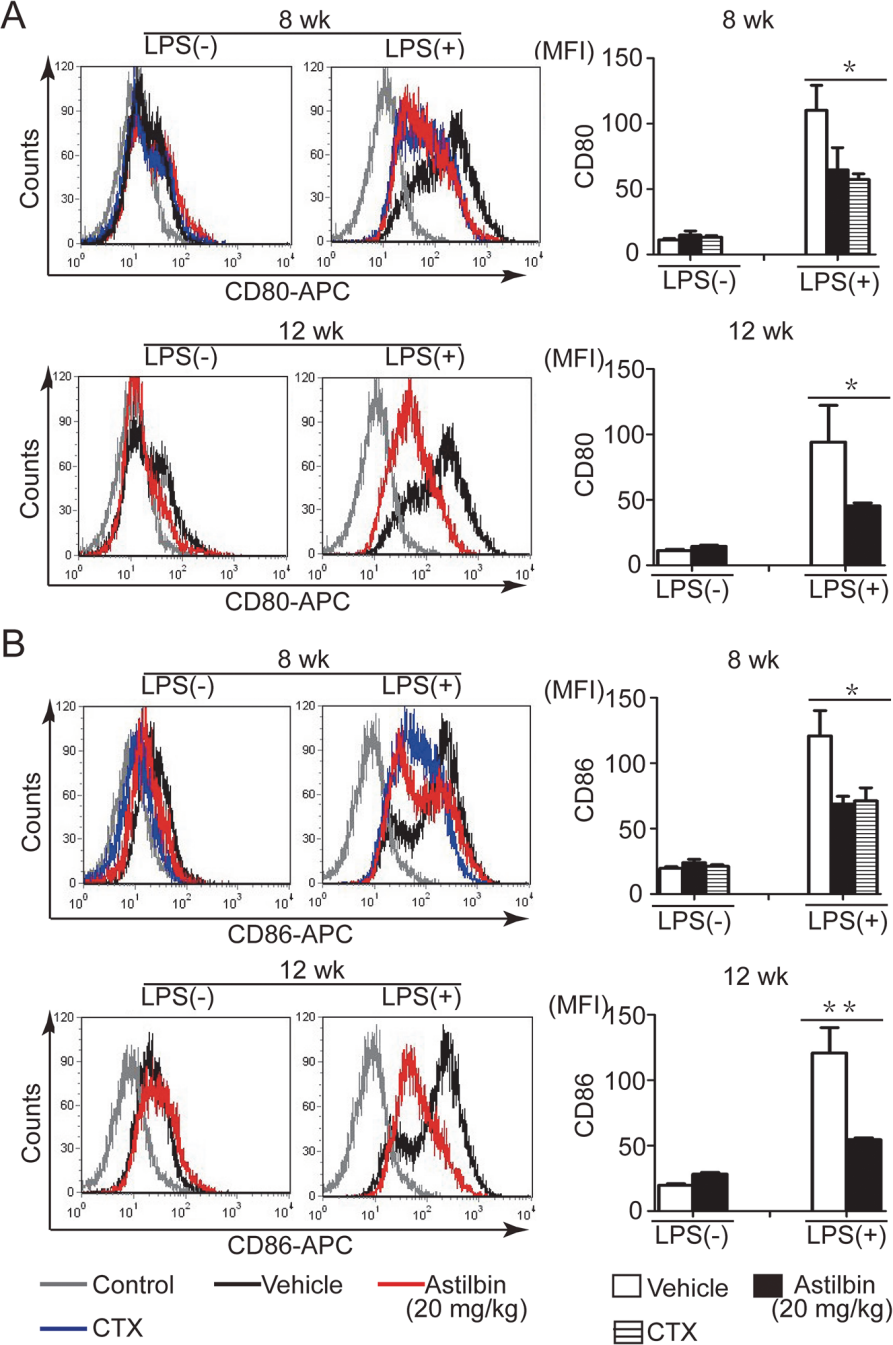
Astilbin treatment decreases CD80 and CD86 expression on spleen B cells from MRL/lpr mice. MRL/lpr mice were treated with astilbin starting at 8 or 12 weeks of age. At 20 wk, splenocytes were isolated from the mice and stimulated with LPS (1 μg/ml) or PBS for 24 h. Left panel, representative histograms comparing CD80 (**A**) and CD86 (**B**) expression gated for CD19^+^ cells. Right panel, cumulative data of flow cytometry for CD80 (**A**) and CD86 (**B**) expression gated for CD19^+^ cells. MFI: mean fluorescence intensity. Gray line represents isotype control staining. The data show mean ± SEM, n = 3, 3, 4 mice for vehicle, astilbin, CTX treatment, respectively. * *p* < 0.05,** *p*< 0.01.

Next, the impact of astilbin treatment on the antigen-presenting function of activated B cells was tested. The spleens of treated MRL/lpr mice were isolated and stimulated with LPS. *Ex vivo* experiments showed that the 20 mg/kg dose of astilbin significantly inhibited the upregulation of the co-stimulatory molecules CD80 and CD86 on CD19^+^ B cells in response to LPS when administered from either 8 or 12 wk (Fig 7A and 7B). These results suggest that astilbin treatment decreases B cell capacity to stimulate T lymphocytes.

### In vitro effect of astilbin on CD80 and CD86 expression in activated B cells

**Fig 8:**
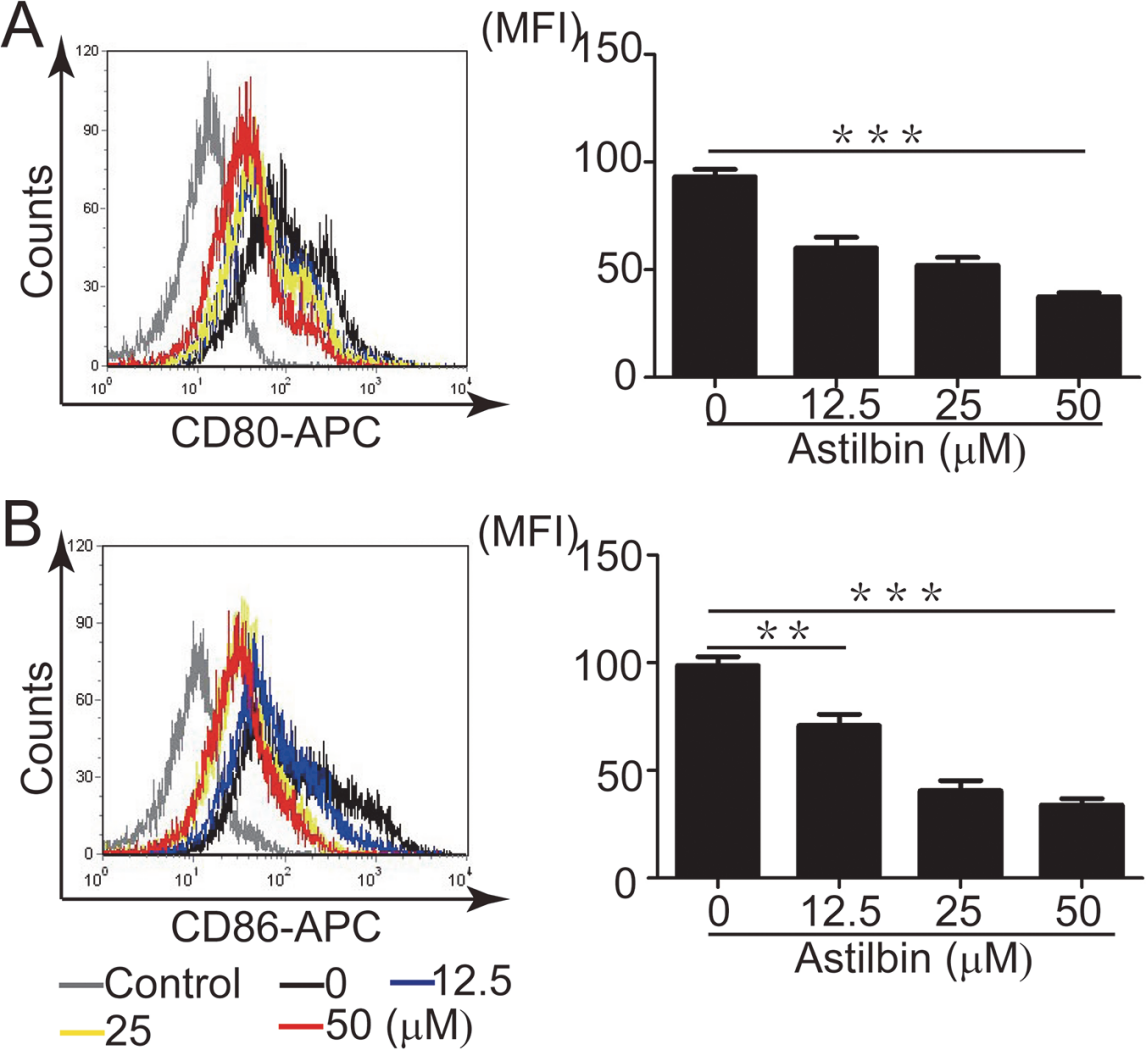
Astilbin downregulates CD80 and CD86 expression on activated B cells. Splenocytes were isolated from naive MRL/lpr mice and stimulated with LPS (1 μg/ml) in the presence of various concentrations of astilbin for 24 h. Left panel, representative histograms comparing CD80 (**A**) and CD86 (**B**) expression gated for CD19^+^ cells. Right panel, cumulative data of flow cytometry for CD80 (**A**) and CD86 (**B**) expression gated for CD19^+^ cells. MFI: mean fluorescence intensity. Gray line represents isotype control staining. The data show mean ± SEM of at least three mice. ** *p* < 0.01,** * *p*< 0.005.

To further investigate whether astilbin directly downregulates CD80 and CD86 expression on activated B cells, we isolated the splenocytes from naïve MRL/lpr mice and stimulated them with LPS in the presence of various concentrations of astilbin for 24h. Astilbin reduced expression of these co-stimulatory molecules on CD19^+^ B cells in a dose-dependent manner (Fig 8).

## Discussion

Although survival of patients with SLE has greatly improved compared to earlier decades, this improvement appears to have reached a plateau. Patients with SLE continue to have mortality risk greater than three times higher than the general population [26]. Controlling disease activity, minimizing the use of corticosteroids and optimizing management of comorbidities have been identified as treatment goals of SLE. An orally available drug taken for a long time to relieve SLE-associated symptoms and possibly slow the progression of the ongoing disease would be advantageous and improve patient compliance with therapy. This study demonstrates that oral administration of a natural flavonoid astilbin, isolated from traditional medicinal herbs, ameliorated SLE-like disease in lupus-prone MRL/lpr mice, accompanied by decreases in the amount of autoantibodies and several SLE-associated proinflammatory cytokines. Most importantly, this natural product shows effectiveness when administered prior to the initiation of the disease, and similar but less profound effectiveness in the mice with the established disease, suggesting the potential of astilbin in treating autoimmune diseases such as SLE.

SLE is best characterized by the presence of activated T and B cells in conjunction with the development of many different autoantibodies and chronic inflammation as clinical symptoms. Similarly, accumulation of activated T and B cells contributes to SLE-like disease in MRL/lpr mice due to a defect in Fas-mediated cell death of T cells [25, 27]. In our study, repeated administration of astilbin starting at 8 wk of age reduced the mass of both spleen and lymph nodes to the level of that of CTX control at the lowest dose of 20 mg/kg. Accordingly, the number of total CD3^+^ T and CD19^+^ B cells also decreased significantly upon astilbin treatment. It is important to note that spleen CD3^+^CD44^hi^CD62L^lo^ activated T cell proportions (including CD4^+^ T and CD8^+^ T cells) greatly declined in the MRL/lpr mice with astilbin treatment initiated at either 8 or 12 wk of age. In contrast, CD3^+^CD44^lo^CD62L^hi^ naïve T cell levels seemed to increase upon treatment (Fig 4A and S2A Fig). It is possible that facilitating apoptosis in activated T cells of astilbin could be responsible for the partial depletion of these pathogenic cells in the MRL/lpr mice. Its unique capability to selectively induce apoptosis in activated but not nonactivated T cells mainly though mitochondria—associated apoptotic pathways have been evidenced by many our previous results [14, 16, 17]. Indeed, astilbin decreased the mitochondrial membrane potential in activated T cells from naïve MRL/lpr mice, supported by an increase in the green fluorescence of JC-1. A recent study has also indicated that Fas-independent T-cell apoptosis maintains peripheral tolerance and thus controls autoimmune arthritis in MRL/lpr mice [28].

Multiple cytokines have been implicated in playing key roles during the initiation, progression, and development of murine and human lupus including, but not limited to, IFN-, IL-17, IL-6, IL-1 and TNF-′ [29–31]. We found that the serum levels of these cytokines were decreased after long-term administration of astilbin. Meanwhile, the mRNA expression levels of IFN-, IL-17A, and TNF-′ were greatly downregulated in the spleens from astilbin-treated mice, indicative of inhibition of T cell function. Reduction or modulation in these cytokine profiles can provide a favorable benefit for patients and provides an indirect indicator of disease resolution. Because IL-6 and IL-17 are essential for B cell activation, survival and differentiation to plasma cells [32, 33], we investigated the impact of astilbin on plasma cell differentiation. Preventive astilbin administration resulted in a decrease in spleen CD19^-^ B220^-^CD138^+^ plasma cells. This finding corresponded well with the reduced levels of serum anti-nuclear antibodies, although this reduction seemed not to persist until 30 wk. In addition, early treatment resulted in a decrease in the splenic mRNA level of BAFF, which is a critical survival factor for transitional and mature B cells and is a promising therapeutic target for SLE [34]. Unlike CTX, astilbin failed to decrease the mRNA expression of APRIL (a proliferation-inducing ligand) or AID (activation-induced deaminase) in the spleen (S3 Fig), suggesting that the mechanism underlying which astilbin acts on B cells is distinct from that of indiscriminate immunosuppressant CTX.

Further *ex vivo* experiments demonstrated that astilbin treatment resulted in significant downregulation of the expression of CD80 and CD86 molecules on B cells stimulated with LPS. Previous studies examining the role of B cells as autoantigen presenting cells (APCs) in the activation of autoreactive T cells, demonstrated that expression of CD86 and/or CD80 molecules by B cells are essential for breaking T cell tolerance to self antigens [35]. Absence of CD86 and/or CD80 co-stimulation interferes with the spontaneous activation and accumulation of memory T lymphocytes in MRL/lpr mice and the development of nephitis and antibody production [30, 36]. *In vitro* experiments demonstrated the direct inhibitory effect of astilbin on their expression in LPS activated B cells, whereas astilbin had no effect on B cell proliferation (data not shown). Therefore, it is inferred that astilbin could also negatively regulate B cell function and humoral immune responses though its effect on CD86 and CD80-mediated signaling in addition to its action on activated T cells.

Taken together, our results indicate that distinct from the present indiscriminate immunosuppressants or special targeted agents for certain cell type, astilbin significantly mitigated disease development in lupus-pronSe mice by partially depleting functional activated T and B cells. This potent, orally active, natural immunosuppressant would take advantage to treat the debilitating clinic-pathological manifestations of SLE.

## Supporting Information

S1 Fig HPLC analysis of astilbin. (TIF)

S2 Fig Astilbin treatment decreases the percentage of CD44^hi^CD62L^lo^ activated CD8^+^ T cells in the spleen. (TIF)

S3 Fig Astilbin treatment had no effects on the mRNA expression of APRIL and AID. (TIF)
