# Supplementary figures and images for: Decrease of Functional Activated T and B Cells and Treatment of Glomerulonephitis in Lupus-Prone Mice Using a Natural Flavonoid Astilbin
Source: PLoS One. 2015 Apr 13;10(4):e0124002. doi: 10.1371/journal.pone.0124002 (PMC4395080; doi:10.1371/journal.pone.0124002)

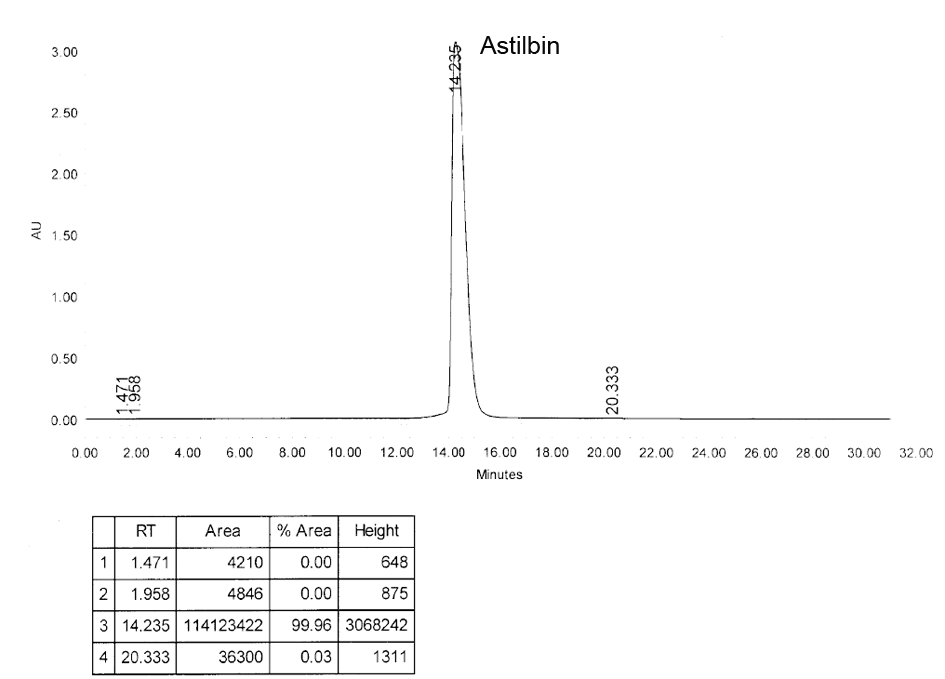

Supplement: S1 Fig — (TIF) [file pone.0124002.s001.tif]

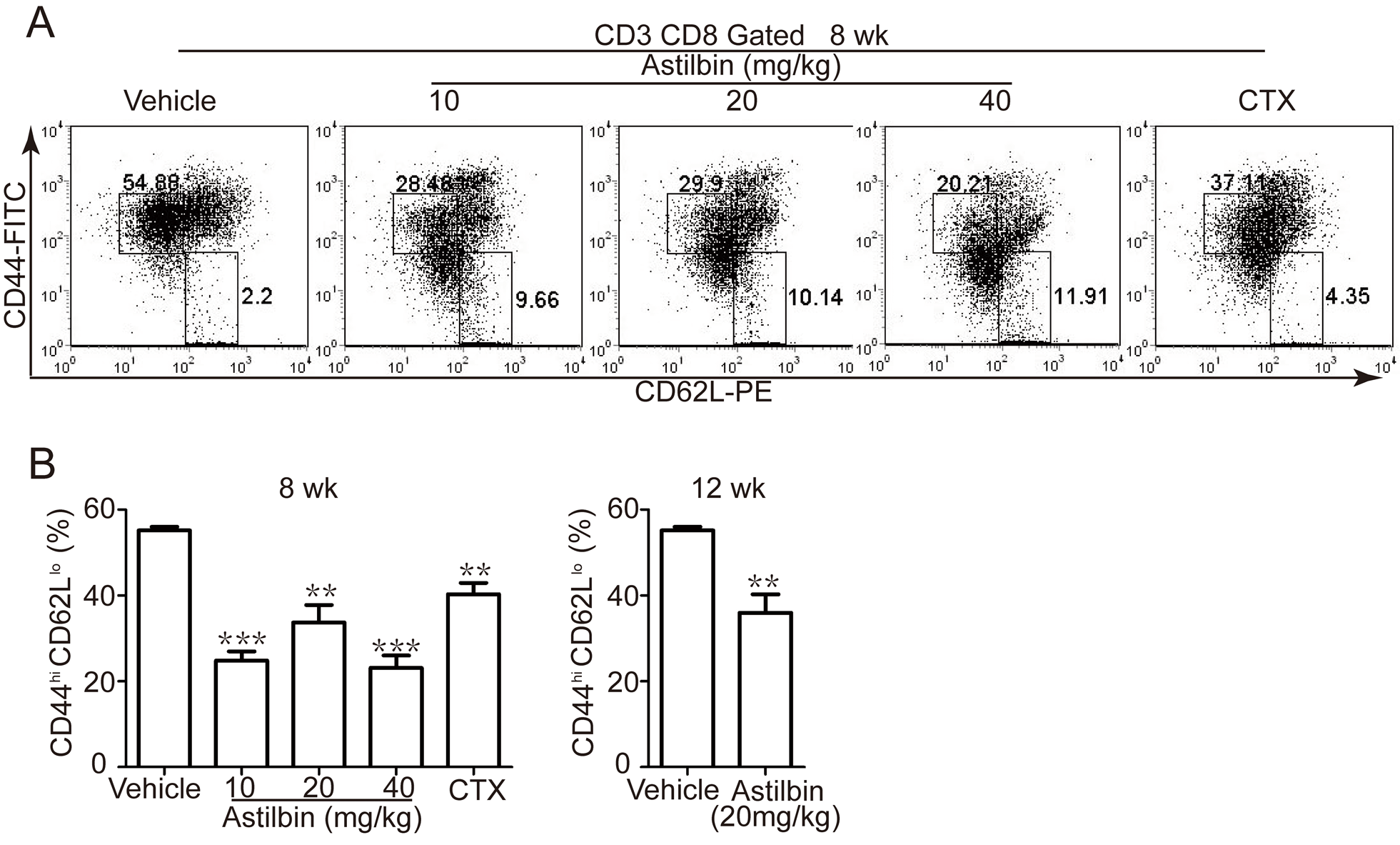

Supplement: S2 Fig — (TIF) [file pone.0124002.s002.tif]

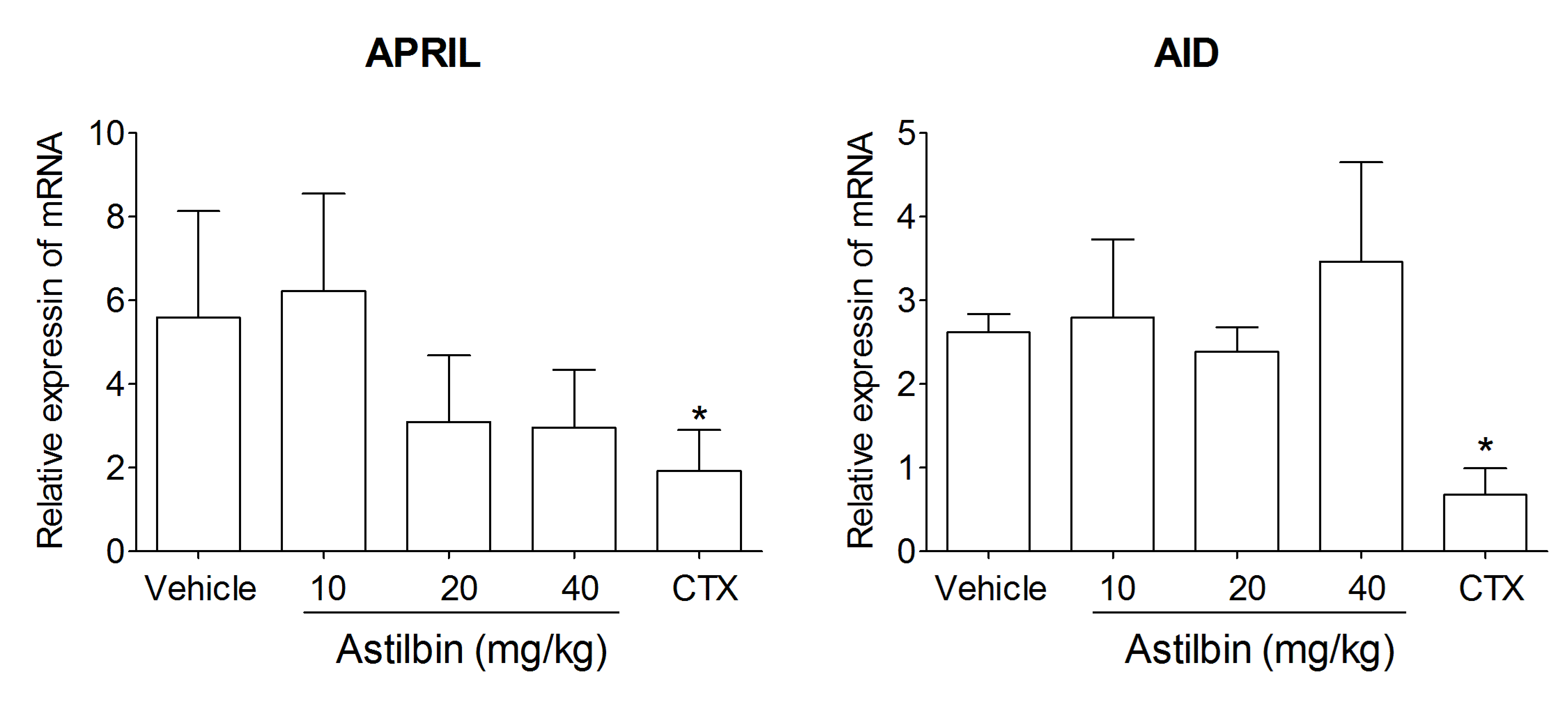

Supplement: S3 Fig — (TIF) [file pone.0124002.s003.tif]
